# Consumer Smartwatch Technology in Health and Performance Research: Validity, Limitations, and Real-World Applications

**DOI:** 10.3390/s26144486

**Published:** 2026-07-15

**Authors:** Adam S. Lepley, Fiddy Davis, Amanda C. Melvin, Zheng-Yang Zhao

**Affiliations:** 1 School of Kinesiology, University of Michigan, Ann Arbor, MI 48109, USA; acmelvin@umich.edu (A.C.M.); zzyang@umich.edu (Z.-Y.Z.); 2 Department of Kinesiology, Division of Social Sciences, Hope College, Holland, MI 49423, USA; davisf@hope.edu

**Keywords:** smartwatch, wearable technology, digital health, sensor-derived metrics, validity, photoplethysmography, physical activity monitoring, health data, data interoperability, real-world monitoring

## Abstract

**Highlights:**

**What are the main findings?**

- Smartwatch-derived metrics should not be interpreted uniformly as direct physiological measurements, as many are modeled estimates generated from sensor signals, proprietary algorithms, user characteristics, and contextual assumptions.
- The validity of smartwatch-derived outcomes varies substantially by metric, device, population, activity type, environment, and intended use, with stronger support for some within-person trend monitoring applications than for precise clinical or physiological quantification.

**What are the implications of the main findings?**

- Researchers and practitioners should evaluate smartwatch data according to the underlying sensor signal, algorithmic transformation, construct validity, ecological context, and intended decision before applying these metrics in health, rehabilitation, or performance settings.
- Transparency, standardized reporting, harmonized data infrastructure, and rigorous validation across diverse populations and real-world conditions are needed to support responsible use of smartwatch-derived data.

**Abstract:**

Consumer smartwatches are increasingly used to monitor health, physical activity, rehabilitation, and performance in real-world environments. Although these devices provide continuous and scalable data, many user-facing outputs are not direct physiological measurements, but estimates generated from sensor signals, proprietary algorithms, user characteristics, and contextual assumptions. This review article provides a practical framework for evaluating smartwatch-derived metrics by distinguishing between relatively direct sensor measurements and higher-level algorithmic outputs. We review how common and emerging metrics are generated, including cardiovascular measures, energy expenditure, aerobic capacity, sleep and readiness scores, body composition, movement mechanics, cuffless blood pressure, sweat loss and hydration, and non-invasive glucose monitoring. Across these domains, validity varies substantially by device, algorithm, population, activity type, environment, and intended application. Smartwatch-derived data may be most useful for tracking within-person trends and complementing laboratory, clinical, or self-reported assessments, but caution is warranted when using these outputs for precise physiological quantification, diagnostic classification, or cross-device comparisons. Future progress will require stronger validation frameworks, greater algorithmic transparency, standardized reporting, harmonized data infrastructure, and careful alignment between wearable metrics and meaningful health, rehabilitation, and performance decisions.

## 1. Introduction

Consumer interest in wearable devices has grown rapidly over the past decade, with nearly one in three Americans reporting regular use [1]. Among these technologies, smartwatches have emerged as one of the most widely adopted devices for monitoring health, activity, and performance over time. Their popularity is driven in part by their multifunctional design, which supports both everyday functions, such as messaging, phone calls, navigation, and smartphone connectivity, and physical activity monitoring through embedded sensors that capture and report physiological and behavioral data in real-world environments. As a result, smartwatches are increasingly being used across a broad range of contexts, including clinical research, population health monitoring, athletic performance tracking, rehabilitation, and personalized health management [2]. Their widespread use and ability to collect repeated, longitudinal data outside of laboratory or clinical settings represent an important shift in how human health, behavior, and performance can be quantified.

Despite this premise, the application and interpretation of smartwatch-derived outcomes remain challenging [3]. Unlike many traditional laboratory-based assessments, most smartwatch outputs are not direct physiological measurements. Instead, most user-facing metrics are predicted values generated by proprietary algorithms applied to raw sensor signals [4,5]. For example, metrics such as energy expenditure, aerobic capacity, sleep staging, body composition, and more abstract constructs such as “readiness” or “recovery” scores are inferred through proprietary models that are often not transparent to researchers, clinicians, or users. Devices that report the same metric may rely on different sensors, sampling strategies, algorithmic assumptions, and validation standards, creating uncertainty about whether a given smartwatch output accurately reflects the physiological or behavioral construct it is intended to represent.

This distinction is central to the appropriate use of wearable technology. The value of a smartwatch-derived metric lies in its validity, reliability, and interpretability, rather than its availability or ease of collection. For example, a metric that is useful for tracking within-person change over time may not be appropriate for diagnostic classification, cross-sectional comparisons between individuals, or clinical decision-making. Similarly, a metric that performs well under controlled laboratory conditions may demonstrate reduced accuracy in real-world settings where movement artifact, device placement, environmental conditions, user behavior, and individual characteristics influence measurement quality. The rapid expansion of wearable capabilities has, in many cases, outpaced the development of methodological frameworks needed to evaluate their performance systematically.

Therefore, the purpose of this review is to provide a practical framework for evaluating consumer smartwatch technology in health, exercise, rehabilitation, and performance research. Specifically, we discuss how smartwatch-derived metrics are generated, how direct and algorithm-derived measurements should be interpreted, and how validity, reliability, physiological relevance, and real-world context should guide the interpretation and application of wearable data. We then review key considerations related to sensor-derived outcomes, data interoperability, applied use cases, and future directions for smartwatch-enabled research and practice. Ultimately, this review aims to help researchers and practitioners move beyond asking what smartwatches *can* measure toward considering what *should* be measured, *why* it matters, and *how* those measurements can be used responsibly to improve human health and performance.

### Review Approach

This article was developed as a narrative review to synthesize and interpret evidence across the broad and rapidly evolving field of smartwatch technology. Because multiple systematic reviews and meta-analyses exist that have already evaluated the objective accuracy of consumer wearable devices, our goal was not to duplicate those efforts, but rather to integrate and translate the existing evidence into an applied framework for interpreting smartwatch-derived metrics. Relevant literature was identified through iterative, topic-focused searches of PubMed, SPORTDiscus, and Google Scholar through June 2026. Search terms included combinations of smartwatch, wearable technology, validation, accuracy, reliability, sensor, photoplethysmography, inertial measurement unit, heart rate, heart rate variability, oxygen saturation, energy expenditure, maximal oxygen uptake, sleep, readiness, body composition, movement mechanics, blood pressure, hydration, sweat loss, glucose, interoperability, and digital health. Searches were supplemented by reference-list screening and targeted review of regulatory documents, manufacturer technical documentation, and professional or consensus guidance when these sources were needed to describe device operation, data access, or current regulatory status.

## 2. Conceptual Framework for Wearable-Derived Measurements

A fundamental challenge in evaluating consumer wearable devices is determining what is actually being measured, how a metric is generated from raw data, and whether that metric appropriately represents the intended physiological or behavioral construct. The presence of a device-generated value does not necessarily mean that the value is valid, interpretable, or useful for a given application. Unlike many laboratory-based instruments, which are designed to directly assess specific physiological or biomechanical variables, smartwatches typically rely on indirect measurements that are transformed into user-facing metrics through multiple layers of signal processing and algorithmic modeling [6]. To interpret these outputs appropriately, wearable measurement should be understood as a multi-stage process.

**Figure 1:**
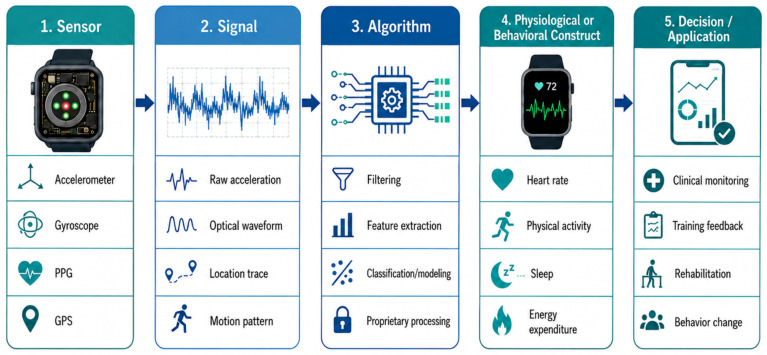
Conceptual framework for smartwatch-derived measurement. Smartwatch-derived metrics are generated through a multi-stage process in which embedded sensors capture raw data, signals are processed and transformed through algorithms, and outputs are interpreted as physiological or behavioral constructs that inform health, performance, rehabilitation, or behavior-change decisions. Smartwatch outputs should be evaluated according to analytical validity, algorithmic validity, construct validity, and ecological validity before being applied in research or practice. Created in BioRender under an appropriate publication license. Lepley, A. (2026) https://BioRender.com/urzhel7 (accessed on 10 July 2026). BioRender AI-assisted tools were used to support figure layout and visual refinement within the BioRender platform. The authors reviewed, edited, and approved the final figure and take full responsibility for its content and accuracy.

At a basic level, smartwatches function as systems that convert data from embedded hardware sensors into processed signals, transform those signals through algorithms, and present derived outputs intended to inform user, clinical, research, or performance decisions. Sensors such as accelerometers, gyroscopes, global positioning systems (GPS), and photoplethysmography (PPG) sensors collect raw data related to device motion, orientation, location, or local physiological signals at the point of wear. These raw data are then processed into signals that are intended to represent user-level movement, behavioral activity, or physiological function. In many cases, these signals are further interpreted through proprietary algorithms to estimate higher-level constructs such as energy expenditure, sleep stage, aerobic fitness, body composition, or recovery status. Finally, these derived metrics are used to guide decisions related to health, performance, rehabilitation, or behavior. This progression can be conceptualized as a pathway from sensor to signal to algorithm to physiological or behavioral construct to decision (Figure 1).

Within this framework, it is important to distinguish between direct sensor measurements and derived algorithmic outputs. Raw acceleration, angular velocity, location, or optical signal data are closer to the physical phenomena captured by the device [7,8]. In contrast, most smartwatch-derived metrics represent modeled estimates that depend on assumptions embedded within device-specific algorithms. These assumptions may vary across manufacturers, device models, software versions, and populations and are not fully transparent to researchers or users. Evaluating smartwatch-derived metrics therefore requires consideration of several levels of validity. Analytical validity refers to how accurately the sensor captures the underlying signal, such as whether a PPG sensor accurately detects pulsatile blood volume changes at the wrist. Algorithmic validity reflects how accurately the device converts raw or processed signals into a derived output, such as transforming a PPG waveform into an estimated heart rate. Construct validity addresses whether the derived output adequately represents the intended physiological or behavioral construct, such as whether PPG-derived heart rate reflects true cardiac heart rate. Lastly, ecological validity considers whether the measurement performs adequately in real-world environments, where motion artifact, device placement, skin tone, sweat, temperature, exercise intensity, and user behavior may influence signal quality and algorithm performance.

This multi-level review highlights several considerations for the interpretation of wearable data. First, the rapid expansion of available metrics may encourage over-measurement, in which users monitor variables simply because they are available rather than because they are valid or meaningful [9]. Second, derived outputs may be misinterpreted as direct physiological measurements, leading to inappropriate conclusions about health, performance, or recovery [10,11]. Third, measurement accuracy is often context-dependent and may vary by activity type, intensity, environment, wear location, device fit, and individual characteristics such as age, sex, skin tone, body composition, fitness level, or clinical status [12,13].

The utility of smartwatch technology should therefore not be defined by the number of metrics a device provides, but by how well those metrics align with the intended purpose. Establishing a conceptual framework for wearable measurement is essential for determining when smartwatch-derived data can be used confidently, when caution is warranted, and when additional validation or complementary assessment is needed. This framework provides the foundation for evaluating smartwatch-derived metrics throughout the remainder of this article. Rather than asking whether a smartwatch can generate a given output, researchers and practitioners should critically evaluate what sensor signals underlie the metric, how those signals are processed, what physiological or behavioral construct is being inferred, and whether that inference is appropriate for the intended research, clinical, performance, or health-related decision.

## 3. Beneath the Watch Face: Sensors and Signal Integration in Smartwatches

With this measurement framework in mind, it is important to consider the hardware systems that enable smartwatch-derived metrics. Understanding the primary sensors within smartwatches provides a foundation for evaluating what these devices can measure directly, what they predict indirectly, and where error may be introduced.

At the center of most smartwatch systems is a microcontroller or embedded processing unit that coordinates sensor sampling, stores data, and communicates with paired smartphones or cloud-based platforms [14]. The microcontroller does not directly measure physiology or behavior, but it enables synchronized data acquisition across multiple sensors and supports real-time processing of raw signals into interpretable outputs. In this way, the smartwatch functions less as a single measurement device and more as a compact, multi-sensor computing platform.

Motion sensing is primarily accomplished through inertial measurement unit (IMU) components, which typically incorporate accelerometers, gyroscopes, and magnetometers [15]. Accelerometers detect linear acceleration and are commonly used to estimate step counts, movement intensity, sleep-wake patterns, and activity classification [7]. Gyroscopes measure angular velocity and provide information about rotation and orientation, which can improve the detection of complex movements, postural changes, or sport-specific movement patterns [16]. Magnetometers estimate orientation relative to the Earth’s magnetic field and may support direction, navigation, and contextual movement interpretation [17]. In addition, GPS sensors are often integrated with IMU components and provide location-based data that are commonly used to estimate distance, pace, speed, elevation change, and route characteristics during outdoor activities [18]. Collectively, these sensors provide information about device movement that is then extrapolated to infer human movement.

Optical sensors are central to smartwatch-based physiological monitoring. Most consumer smartwatches use PPG, an optical sensing technique that uses light-emitting diodes (LEDs) to emit specific wavelengths into the skin and photodetectors that measure changes in reflected light associated with microvascular blood volume changes [19]. PPG is most commonly used to estimate heart rate (green light wavelengths, approximately 530 nm) and also contributes to the estimation of other cardiovascular outputs such as heart rate variability (HRV) and oxygen saturation (red, approximately 660 nm; infrared, about 940 nm) [8,19,20]. Although PPG can provide useful physiological estimates under stable conditions, signal quality may be sensitive to motion artifact, skin-device contact, sensor placement, tissue characteristics, skin tone, ambient light, sweat, temperature, and exercise intensity [8,20,21,22]. Thus, output metric accuracy depends on both the quality of the captured signal and the algorithms used to interpret that signal.

Some smartwatches also incorporate additional sensors that provide physiological or environmental context. Barometric altimeters may estimate elevation change or stair climbing [23], temperature sensors may capture skin or environmental temperature [24], and electrical sensors may support single-lead electrocardiogram recordings [25] or bioelectrical impedance-based estimates of body composition in select devices [26]. Although these sensors expand the application capability of smartwatches, they also introduce additional assumptions, sources of error, and context-specific limitations. Each sensor must therefore be evaluated according to the construct it is intended to support and the conditions under which the measurement is collected.

Importantly, smartwatches should be understood as multi-sensor fusion devices rather than single-measure tools. Many common outputs are not derived from one sensor alone, but from the integration of multiple sensor streams combined with user characteristics and proprietary algorithmic assumptions [13]. For example, energy expenditure estimates may incorporate acceleration, heart rate, demographic characteristics, and activity classification models [11,27]. Sleep estimates may combine movement, heart rate, HRV, timing, and behavioral assumptions [28,29]. Aerobic fitness estimates may integrate heart rate responses, walking or running speed, GPS-derived pace, and user-reported characteristics [4,30]. Similarly, training load or recovery-related outputs may combine multiple physiological and behavioral inputs into a single composite score [31].

### Device Categories

Although consumer wrist-worn wearables are often discussed collectively, available devices can differ substantially in sensor configuration, intended use, processing capacity, battery life, data accessibility, and supporting validation evidence [32,33,34,35]. There is currently no universally accepted scientific taxonomy distinguishing consumer wrist-worn devices by capability; however, available devices can be pragmatically grouped as activity or fitness trackers, general-purpose smartwatches, and performance-oriented premium models. These categories overlap and should therefore be viewed as descriptive rather than fixed classifications. Wrist-worn activity trackers generally prioritize affordability and relatively basic outcomes such as step count, heart rate, activity intensity, and sleep duration. Their lower cost may support large-scale or longitudinal studies, but some models provide fewer sensors, limited onboard processing, reduced access to high-resolution data, or reliance on a paired smartphone for location-based measurements. General-purpose smartwatches combine activity monitoring with touchscreen interfaces, extensive smartphone integration, and broader health-oriented sensors and outcomes. Depending on the model and software version, these devices may incorporate some or all of the hardware sensors discussed above. Their strengths include broad consumer adoption, frequent software development, extensive multisensor integration, and the availability of selected health outcomes. Limitations, however, include more frequent charging, operating-system dependence, proprietary processing, and variable access to raw or high-resolution data [3,12,36]. Performance-oriented sport watches, typically marketed as “Ultra” or “Premium” models, generally emphasize extended battery life, enhanced outdoor navigation and positional data, sport-specific activity profiles, and proprietary training, recovery, and workload metrics. These features may make them well suited for prolonged exercise monitoring, outdoor activity, and athlete-management applications. However, the presence of additional or more sophisticated outputs does not necessarily indicate stronger criterion validity, and performance still varies by device, metric, activity, population, and testing condition [3,12,13]. Consequently, device selection should be based on the specific outcome, intended population, activity, environment, monitoring duration, required sampling resolution, data-access pathway, and intended decision to be supported.

Regardless of the specific device, multi-sensor fusion is both a strength and a limitation of smartwatch technology. By integrating multiple data streams, smartwatches can provide richer context than any individual sensor alone. More advanced multimodal fusion may further improve robustness by combining complementary physiological, inertial, positional, and environmental signals and dynamically weighting them according to signal quality and context. For example, integration of PPG or ECG with accelerometry, gyroscopy, satellite positioning, and pedestrian dead reckoning may allow motion or location estimates to remain available when an individual sensor becomes unreliable [37,38]. Recent wrist-worn multimodal systems demonstrate the technical feasibility of combining health monitoring and pedestrian navigation within a unified platform, although independent validation across diverse users and real-world conditions remains necessary [37]. Such approaches could strengthen ecological validity by reducing dependence on any single sensor and improving continuity across indoor, outdoor, resting, and active conditions.

Although multisensor fusion can provide richer and more robust information than any individual sensor alone, the resulting metrics still depend on proprietary algorithms, signal-weighting strategies, and modeling assumptions. Consequently, their accuracy and interpretability may vary across devices, populations, activities, and environments. Researchers and practitioners should therefore evaluate smartwatch-derived metrics not only according to the sensors included in the device, but also according to how those signals are processed, integrated, validated, and applied to the intended research, clinical, performance, or health-related question.

## 4. What Is the Watch Actually Measuring? Detailed Evaluation of Sensor-Derived Metrics

The preceding sections establish a framework for understanding smartwatches as multi-sensor systems that transform raw signals into user-facing physiological and behavioral metrics. To apply this framework, it is necessary to examine how specific smartwatch-derived outcomes are generated, what they are actually measuring, and where error may be introduced. Smartwatch-derived metrics vary substantially in how closely they approximate the physiological or behavioral constructs they are intended to represent. Some outputs are derived from relatively direct sensor signals, while others are higher-level estimates generated through proprietary algorithms that combine multiple sensor inputs and user characteristics. Therefore, the validity and interpretability of smartwatch-derived metrics should be evaluated according to the underlying signal being captured, the algorithm used to transform that signal, and the context in which the metric is applied. The following section applies this approach to several commonly reported and emerging smartwatch metrics, moving from cardiovascular and metabolic outcomes to sleep, body composition, movement mechanics, and emerging physiological estimates. These metrics are also highlighted in Table 1.

### 4.1. Cardiovascular Metrics

As described above, smartwatch estimates of heart rate and peripheral oxygen saturation (SpO_2_) rely primarily on PPG. When validated against gold-standard criterion measures (multi-lead electrocardiography (ECG) for heart rate and arterial blood gas for SpO_2_), consumer wearables demonstrate acceptable fidelity under resting and steady-state conditions, with mean absolute percentage error (MAPE) values generally below 10% and evidence of improved performance in newer device models [39,40,41,42]. However, during dynamic or transitional physiological states, such as changes in exercise intensity, validation accuracy declines, with peripheral pulse rate lagging behind electrical cardiac activity [43,44]. The accuracy of PPG-derived cardiovascular metrics also declines when signal quality is degraded by motion artifact, poor device fit, or skin-device interference from tattoos, hair, or individual differences in skin tone [21]. Epidermal melanin content represents an important equity and accuracy concern for optical wrist-worn wearables because melanin can attenuate light transmission at certain wavelengths. For green-light PPG, this may reduce signal-to-noise ratio and contribute to heart rate underestimation, whereas for red and infrared wavelengths used in SpO_2_ estimation, melanin-related differences in light absorption may bias the red/infrared ratio and contribute to oxygen saturation overestimation in individuals with darker skin tones [45,46,47]. This persistent bias prompted the U.S. Food and Drug Administration (FDA) to issue updated guidance in 2025 requiring skin tone subgroup reporting in pulse oximeter validation submissions [45,48]. Demographic and anthropometric characteristics beyond skin tone may also affect smartwatch-derived heart-rate accuracy. Age-related differences in vascular compliance, peripheral perfusion, cardiac rhythm, and movement patterns may influence PPG signal quality, while higher body mass index, adiposity, and wrist circumference may affect optical transmission and device-to-skin contact. Sex-related differences in wrist anatomy, vascular physiology, and heart-rate responses may introduce additional variability. Thus, available evidence suggests that heart rate validity can differ across age, body mass index, and sex groups, although the direction and magnitude of these effects vary across devices, activity conditions, and study populations, rather than producing a consistent bias across all wearables [49,50]. Therefore, validation findings should not be assumed to generalize across demographic groups unless subgroup performance has been explicitly evaluated and reported.

HRV is also reported by consumer smartwatches; however, it is not measured in its classical electrophysiological sense. Whereas clinical HRV is derived from the beat-to-beat R–R intervals of ECG, optical wearables derive an analogous metric, pulse rate variability (PRV), from the time intervals between successive systolic peaks of the PPG waveform [51]. These devices generate an inter-beat time series interval from which variability indices are calculated. Because robust HRV computation depends entirely on precise peak localization, manufacturers apply automated peak-detection and signal-quality algorithms to reject noisy segments before deriving the inter-beat intervals [52]. The sampling rate of the optical sensor constrains achievable accuracy. For example, a PPG sampling frequency of roughly 100–200 Hz is required to keep PRV index bias below 2%, whereas sampling at 40–50 Hz inflates bias beyond 20% [53]. An important conceptual caveat is that PRV is not strictly interchangeable with HRV, as the pulse wave arrives at the periphery after a variable pulse transit time. In addition to the limitations of PPG signal quality discussed above, reported agreement between PRV and ECG-derived HRV varies across devices, populations, and recording conditions [54,55]. Therefore, PRV should not be treated as a clinical surrogate for HRV [56]. Accordingly, in the technology’s current state, PRV is best interpreted as a valid tool for tracking longitudinal trends and overall autonomic balance, especially in resting or sleeping states, rather than as a substitute for ECG-derived HRV [56,57].

Certain consumer smartwatch models use a single-lead ECG to record actual electrical activity. The smartwatch establishes a bipolar electrical circuit using two dry electrodes, one consisting of the conductive caseback in contact with the wrist, and a second electrode on the digital crown or a side button that is touched by a finger of the contralateral hand [58]. Completing the circuit across the two arms reconstructs a tracing analogous to Einthoven’s Lead I of the standard 12-lead ECG, capturing the potential difference generated by atrial and ventricular depolarization [59]. Against the gold-standard physician-interpreted 12-lead ECG, the single-lead ECG performs strongly for discriminating atrial fibrillation from sinus rhythm under controlled conditions, with sensitivity and specificity commonly exceeding 90–95% [58,60]. Because single-lead ECG records electrical activity, it is not affected by the same limitations as PPG-derived cardiovascular metrics. However, it remains sensitive to motion and skin-electrode contact quality, so single-lead ECG depends on stable, low-impedance skin-to-electrode contact across the entire recording window, and movement during a traditional 30 s measurement can increase error.

### 4.2. VO_2_max and Energy Expenditure Estimation

Maximal oxygen uptake (VO_2_max) and energy expenditure (EE) are not directly measured by consumer wearables. Instead, both are modeled outputs derived by combining sensor data with user-specific anthropometric inputs through proprietary algorithms, compounding the underlying measurement error with additional modeling assumptions. The predominant approach used by commercial smartwatches to estimate VO_2_max relies on the near-linear relationship between heart rate and external workload during submaximal exercise. During an exercise session, the algorithm pairs GPS-derived speed (external work) with simultaneous heart rate (internal cost) and extrapolates the heart rate-speed relationship toward an estimated maximal heart rate to predict VO_2_max, with the estimate refined across repeated exercise sessions [61]. Reported MAPE values commonly fall in the range of roughly 5–13%, depending on device and population, and estimates track within-person change over time reasonably well [62,63,64]. Accuracy appears greatest in healthy, untrained or moderately trained adults and declines in those with the highest fitness level [65]. Furthermore, as it is conventionally expressed per kilogram of body mass, errors in the body-mass input and in the distribution of that mass between fat and lean tissue directly bias the normalized estimate [4,66].

Consumer wearable estimation of EE commonly uses multi-sensor algorithms that integrate PPG-derived heart rate, motion signals from accelerometers, gyroscopes, GPS, and user-specified characteristics such as age, sex, height, and body mass [67]. The accelerometer signal is typically mapped to metabolic equivalents of task (METs) using either fixed activity-classification cut-points or trained regression and machine-learning models. Heart rate provides an intensity-related adjustment, whereas the resting component is approximated from a basal metabolic rate (BMR) computed from the anthropometric inputs [68]. Because basal metabolism accounts for a substantial proportion of total daily EE, errors in the anthropometric estimation of resting metabolism propagate directly into the reported user output. Multiple systematic reviews have concluded that consumer wearables estimate EE with insufficient accuracy for individual decision-making, with errors at the device level frequently exceeding ±10–20% [40,69]. EE validity is significantly worse in individuals who are overweight or obese compared to normal-weight populations, motivating recent work on explicitly BMI-inclusive EE algorithms [49,68]. During physical activity, devices tend to underestimate EE during low-to-moderate activity but overestimate it at higher exercise intensities, and no major brand has consistently fallen within a ±10% equivalence zone [69,70]. For this reason, wearable EE outputs are best treated as directional indicators of relative effort rather than as accurate caloric output.

Overall, errors in user-entered anthropometric data, motion artifact, activity type, and body composition may propagate through the underlying models and can influence both VO_2_max and EE estimates.

### 4.3. Sleep and Readiness Scores

Polysomnography (PSG) is the criterion method for assessing sleep, and classifies the brain’s electrical activity into wake, rapid eye movement, and non-rapid eye movement stages using electroencephalography (EEG), electrooculography (EOG), and electromyography (EMG) [71,72,73]. Wrist-worn devices have access to none of these neural signals but rather infer sleep structure indirectly from two proxy channels, including gross body movement from a triaxial accelerometer and cardiovascular metrics derived from the PPG sensor. There signales are sometimes supplemented by respiratory rate and skin temperature [72]. Machine-learning algorithms map these proxy signals to patterns of movement and autonomic activity onto sleep stages by combining movement or stillness with heart rate and changes in HRV. Because even expert PSG scoring is imperfect, validation of smartwatch-derived sleep must be interpreted against the limits of the reference itself. For the binary sleep-versus-wake distinction, wearables perform well, with sleep-detection sensitivity commonly at or above 95% and overall agreement exceeding 90% with PSG [74,75,76]. Multi-stage classification is considerably weaker, with sleep-stage sensitivity ranging from roughly 50–86% across stages and devices [75] and a consistent tendency to misclassify wake as light sleep [77]. A recurring systematic bias is that movement-dependent algorithms overestimate sleep and underestimate wakefulness, because a person lying still but awake is read as asleep. Consequently, wearables tend to inflate total sleep time and sleep efficiency while underestimating wake time during sleep interruptions [77,78].

Readiness and recovery scores are intended to provide users with a simplified estimate of how prepared they may be for physical, cognitive, or daily stressors, or how well they may have recovered from recent training, activity, sleep loss, or other accumulated strain. These metrics incorporate yet another layer of abstraction beyond sleep data. Marketed under proprietary names across brands, these composite health scores combine multiple physiological and behavioral inputs, such as overnight resting heart rate, heart-rate variability, sleep duration, recent physical activity, and training load, into a single normalized value partitioned into interpretive zones [79,80]. The weighting and personalization of these inputs against an individual baseline are proprietary and differ substantially between manufacturers. As a result, similar underlying physiological data may be translated into different readiness or recovery scores across devices [79,80]. Although their physiological inputs can be validated, as discussed in the sections above, there is no gold standard against which device-specific scores can be compared. Thus, the composite scores themselves are largely unvalidated. A systematic evaluation of 14 composite health scores across 10 consumer wearable manufacturers found that proprietary weighting schemes generally lack published validation and that theoretical rationale is often emphasized over empirical evidence [79]. Accordingly, in their current state, these scores are best interpreted as algorithmic summaries or interpretations rather than direct physiological measurements. However, with greater transparency, prospective validation, and stronger linkage to meaningful outcomes such as performance, illness, injury risk, or recovery trajectories, these metrics may continue to evolve into more useful decision-support tools.

### 4.4. Body Composition Metrics

Bioelectrical impedance analysis (BIA) differs from the preceding modalities in this document, as it is neither optical, electrocardiographic, nor inertial, but a volume-conduction technique. A small, imperceptible alternating current (commonly on the order of 500–800 µA at 50 kHz) is provided through surface electrodes, and the body’s opposition to that current, termed impedance (Z), is measured [81,82]. Impedance comprises two components: resistance (R, the opposition of body fluids to current flow) and reactance (Xc, the capacitive effect of cell membranes). The physiological basis is that fat-free tissue, being rich in water and electrolytes, conducts current far better than fat [81,83]. Smartwatch-derived BIA extends principles historically used in clinical and research-grade BIA systems into a consumer wrist-worn format. Validation studies of smartwatch-based BIA against criterion standard dual-energy X-ray absorptiometry suggest strong correlations for body fat percentage, but also reveal important limitations, including sex-specific differences in agreement and proportional bias such that error increases among individuals with higher body fat percentages [26,84,85,86,87]. Hydration is the dominant confounder for BIA body composition assessments. Dehydration raises impedance and inflates the apparent fat and skeletal muscle estimates, while fluid overload lowers impedance and results in fat underestimation [81,88]. Due to the sensitivity of BIA measurements to natural everyday variations and conditions such as recent food or fluid intake or a bout of exercise, standardized protocols commonly require fasting, avoidance of recent exercise, and a resting period before measurement for the most accurate results [89,90].

### 4.5. Mechanics: Spatiotemporal, Distance, Velocity, and Movement Metrics

Mechanics metrics are derived from IMU signals that capture device acceleration, rotation, and orientation. Spatial movement, distance, displacement, and velocity are not measured directly, but are computed from these raw signals through sensor-fusion algorithms [91,92]. For outdoor distance and velocity, GPS or other global navigation satellite system signals are typically the primary source. When satellite signals are weak or unavailable, wearable navigation methods may rely more heavily on local inertial data, using accelerometer-derived step detection, estimated step or stride length, and heading information from gyroscopes and magnetometers to propagate position [93,94]. Lacking a foot-mounted sensor to detect gait events at the feet, wrist-worn consumer devices detect steps from the rhythmic acceleration of arm swing and estimate distance by multiplying step count by a modeled stride length derived from user height and cadence, introducing another layer of estimation and potential error [95]. Criterion standards for these metrics are spatial rather than physiological, such as surveyed distances for total distance, radar or timing gates for velocity, and three-dimensional optical motion capture with force plates for gait events [91,96]. Validity of wrist-worn consumer estimates varies by outcome. Wrist devices count steps acceptably in healthy adults at normal walking speeds outdoors but tend to underestimate steps during treadmill walking [97]. Smartwatch-derived metrics such as step count and average cadence generally demonstrate stronger agreement because these outcomes primarily require consistent detection of rhythmic movement patterns at the wrist. By contrast, more complex spatiotemporal variables, including ground contact time and stride length, tend to show poorer agreement since they require accurate identification of discrete gait events, such as foot strike and toe-off, as well as estimation of spatial displacement [98]. Thus, anatomical placement is an important determinant of measurement accuracy. Environmental context also influences the validity of smartwatch-derived movement outcomes. GPS-enabled metrics generally perform better outdoors than indoors, but signal obstruction and multipath error from tall buildings, dense tree canopy, or indoor-adjacent settings can still impact estimates of distance, pace, and velocity [99,100]. These environmental effects may interact with user-entered characteristics, which can introduce additional error, and may systematically influence movement estimates across real-world environments.

Emerging consumer positioning technologies may improve outdoor spatiotemporal measurement beyond conventional single-frequency GPS. Multi-frequency and multi-constellation global navigation satellite system (GNSS) approaches, assisted GNSS, precise point positioning, and map-aided correction methods can reduce initialization time and improve positional accuracy by better correcting atmospheric, clock, and non-line-of-sight errors. Smartphone-based evaluations have demonstrated root mean square positioning errors below approximately 1.5 m in open-sky conditions and 2.5–4.0 m in more challenging real-world environments using assisted precise point positioning approaches [101]. Although these capabilities are not yet uniformly available in smartwatches, their integration into future wrist-worn devices could improve estimates of distance, pace, route, and elevation, particularly in complex outdoor environments.

### 4.6. Emerging Metrics: Cuffless Blood Pressure, Sweat Loss/Hydration, and Glucose Monitoring

The metrics in this cluster are grouped not by a shared sensor but by a shared maturity in deployment in smartwatch devices. Cuffless blood pressure (BP) estimation uses optical-based sensors, but it extracts a different feature of the PPG signal than the metrics discussed earlier. Pulse transit time (PTT) is the interval a pressure wave takes to travel between two arterial sites, and it is inversely related to BP. As pressure rises, the arterial wall stiffens, pulse-wave velocity increases, and transit time shortens [102]. In wearables, this is most often operationalized as pulse arrival time (PAT), or the delay from the R-wave of a single-lead ECG to a point on the PPG waveform at a peripheral site [102,103]. Some single-sensor implementations dispense with the ECG and infer BP from features of the PPG waveform alone [103,104]. Importantly, the device does not measure pressure directly; instead, it measures a timing or morphological proxy and maps it to pressure through a calibration that is specific to the individual and to the moment it was performed. Compared against cuff-based sphygmomanometry or invasive intra-arterial reference measurements, wearable cuffless BP systems have shown variable but sometimes promising agreement. Some studies report relatively small average errors for selected systolic, diastolic, or mean arterial pressure outcomes, but agreement differs by parameter, calibration approach, device model, and study setting. Validation studies report variable accuracy, as several have identified mean errors or mean absolute differences near the accepted 5 mmHg threshold, whereas others show that only a subset of estimates fall within 5 mmHg. For example, in an intensive care unit cohort, the proportion of absolute errors within 5 mmHg ranged from approximately 50% to 85%, highlighting both the promise and current limitations of cuffless blood pressure estimation [103,105,106,107,108]. In July 2025, a cuffless wrist-worn BP system received U.S. FDA 510(k) clearance for over-the-counter home use, with the cleared indications specifying calibration every 24 h to maintain accuracy [109]. Although this clearance represents an important regulatory milestone for a specific use case, expert consensus statements continue to urge caution. A 2025 international position statement and a 2025 American Heart Association scientific statement concluded that no current cuffless technology has demonstrated adequate accuracy for clinical use, and the 2025 AHA/ACC hypertension guidelines recommended against using cuffless devices for the diagnosis or management of hypertension [110,111]. Because accuracy depends on calibration, estimates are expected to be most accurate immediately after calibration and may degrade as arterial physiology changes, such as during exercise, sleep, posture change, or medication use [112,113]. This represents an important limitation because there are also the conditions under which continuous monitoring may be most valuable.

Sweat loss and hydration status are additional emerging smartwatch outputs. Direct sweat-sensing technologies are currently available, but they are more commonly implemented in skin-worn electrochemical or microfluidic patches rather than integrated directly into smartwatches. These patches collect microliter volumes of sweat at the skin surface to estimate local sweat rate based on channel-filling dynamics and to assess electrolyte concentrations through electrochemical analysis [114,115]. In contrast, current smartwatch technology algorithmically estimates sweat loss and hydration metrics using proprietary models that integrate physiological, movement, and environmental inputs such as heart rate, skin temperature, exercise intensity, accelerometry, ambient temperature, and humidity, without directly sampling sweat [116]. As a result, smartwatch-derived sweat loss and hydration metrics should currently be interpreted as modeled estimates rather than direct biochemical or fluid-balance measurements. Available validation studies suggest large errors compared with criterion methods, with MAPE values ranging from approximately 25% to 33%, and agreement appears to vary across sex, type of activity, environment (indoor vs. outdoor), and fitness level [117].

Non-invasive glucose sensing is a future metric that is still in development and not currently available in commercial smartwatches. These approaches aim to estimate blood glucose by using optical PPG or spectroscopy sensors [118,119]. Minimally invasive continuous glucose monitors currently represent the practical clinical benchmark for clinical use, achieving mean absolute relative differences around 8–10% compared to laboratory-assessed plasma glucose [120]. Non-invasive glucose sensing techniques remain largely developmental, with variable validity and accuracy that has not consistently matched the standards of minimally invasive continuous glucose monitoring systems [119,121]. Accordingly, the U.S. FDA has not authorized any smartwatch to measure glucose and has warned against relying on such devices for glucose monitoring [122].

The metric-specific examples above highlight that smartwatch outputs are shaped not only by sensor accuracy, but also by proprietary algorithms, user-specified characteristics, device-specific assumptions, and context-dependent sources of error. These considerations become even more complex when data are exported, aggregated, or compared across devices, platforms and software versions. Thus, after evaluating what individual smartwatch metrics represent and how they are derived, the next challenge is understanding how those outputs move from the device into usable, reproducible datasets.

## 5. From Device to Dataset: Interoperability and Data Infrastructure

As smartwatch-derived metrics are increasingly incorporated into health, performance, and rehabilitation research, a central challenge is determining whether data generated by different devices, platforms, and software ecosystems can be meaningfully compared, combined, and interpreted. A smartwatch produces a device-specific data product shaped by hardware sensors, firmware, sampling strategies, proprietary algorithms, and platform-level processing decisions. Consequently, two devices may report the same metric label, such as heart rate, steps, EE, or sleep duration, while generating those values through different sensor inputs, filtering procedures, aggregation methods, and algorithmic assumptions [12,13]. Identical terminology therefore does not necessarily indicate measurement equivalence.

This issue becomes particularly important when wearable data are used across brands, device generations, populations, or different sites of multicenter studies. Some metrics may demonstrate acceptable within-device consistency for monitoring longitudinal change yet still show limited agreement with criterion measures or other devices [12,123]. Measurement performance may also vary by exercise mode, environment, wear location, and individual characteristics such as age, sex, skin tone, body composition, fitness level, gait mechanics, or clinical status [124,125]. These sources of variability complicate cross-device comparisons and should be considered when designing studies, selecting devices, and interpreting findings.

Data access and infrastructure introduce additional barriers to the use of smartwatch-derived metrics in research. Consumer platforms differ not only in how data are stored, defined, aggregated, and exported, but also in the software development kits (SDKs), application programming interfaces (APIs), permission structures, and data access mechanisms that researchers have available to use [10]. Some platforms allow access to relatively high-resolution sensor or activity-level data, whereas others provide only daily summaries, processed outputs, or proprietary metrics. These platform-specific differences make it difficult to directly compare or combine data across devices, even when the same metric label is used. For example, stride length is a metric that may not be operationalized consistently across platforms; although stride length is typically defined as the distance covered by two consecutive steps, developer documentation indicates that some devices may instead report step length, or the distance covered by a single step [126,127,128]. In addition, firmware, software, or algorithm updates may alter device outputs over time without clear notification or version-level documentation, creating challenges for longitudinal studies and cross-study comparisons [129]. To improve reproducibility, wearable studies should report, when available, the device brand and model, software or firmware version, data source or platform used, SDK or API access pathway, sampling resolution, wear-time criteria, valid-day definitions, missing-data handling procedures, and any analytic transformations applied to the exported data. Ultimately, the value of smartwatch data depends not only on sensor accuracy or metric validity, but also on the infrastructure needed to bring disparate platforms together, harmonize data streams, document processing decisions, and support reproducible interpretation across devices, populations, and study settings. Although these discrepancies cannot be fully eliminated, researchers can mitigate their impact by verifying metric definitions against developer documentation, preserving version-level metadata, and conducting device- or platform-specific validation studies when possible.

## 6. From Monitoring to Meaning: Applications in Research, Health, and Performance

The value of smartwatch technology is greatest when continuous, real-world data are used to answer questions that cannot be fully captured in laboratory or clinic-based settings. In research, smartwatches enable scalable monitoring of physical activity, sleep, heart rate, and other behavioral or physiological patterns across days, weeks, or months. These data have important applications in epidemiology, clinical trials, and remote monitoring, where longitudinal information can provide insight into daily behavior, intervention adherence, recovery trajectories, and population-level health patterns. Additionally, compared with self-reported measures of activity, smartwatch data can provide more objective, time-stamped estimates of movement, sleep, and physiological patterns in daily life, reducing reliance on recall, perception, or reporting bias, particularly when tracking changes in activity levels or recovery over extended periods [130].

In clinical, rehabilitation, and performance contexts, smartwatches may help extend periodic assessments into daily life. Metrics such as steps, moderate-to-vigorous physical activity quantification, heart rate, and sleep duration, can support return-to-activity tracking, behavioral feedback, and monitoring of recovery outside supervised settings. For athletes and physically active individuals, these data may help characterize training consistency, exercise intensity, and recovery patterns. In clinical populations, including individuals after musculoskeletal injury, people with chronic disease, and older adults, smartwatch data may help describe real-world function, activity limitations, and changes in health behavior over time.

A central strength of smartwatches is their ability to bridge the gap between laboratory-based assessments and habitual behavior. Laboratory measures remain essential for establishing criterion values, such as directly measured oxygen consumption, strength, body composition, or gait biomechanics. However, these assessments often capture physiology or performance under controlled conditions at a single point in time. In contrast, smartwatches provide repeated measurements in naturalistic environments, offering ecological context for how individuals behave, recover, and respond to training or rehabilitation outside the laboratory.

For this reason, smartwatch-derived metrics should generally be viewed as complementary to, rather than replacements for, laboratory, clinical, or self-reported measures. Their strongest application may be personalized trend monitoring, including identifying deviations from baseline, changes in habitual activity, responses to intervention, and opportunities for behavior modification. However, these applications require that the selected metric is valid for the population and context of interest, aligned with the intended purpose, and interpreted alongside other relevant clinical, laboratory, or patient-reported information.

## 7. Challenges and Future Directions for Smartwatch Data

A major challenge underlying smartwatch technology is the speed at which devices, sensors, software updates, and proprietary algorithms evolve. Validation evidence generated for one device model or software version may not apply to newer versions, and algorithm updates may alter outputs without clear user notification or documentation. This is particularly important for longitudinal studies, where changes in a metric over time could reasonably reflect true physiological or behavioral change, or simply device-level modification and differences in data processing. Because many smartwatch-derived metrics are generated through closed algorithms, researchers have limited ability to determine how raw sensor signals are processed, weighted, or translated into user-facing outputs.

These challenges are compounded by data overload, limited standardization, and poor cross-device comparability. Smartwatches generate large volumes of data, but more data do not necessarily produce better interpretation. Without clear definitions, harmonized metrics, and transparent processing pipelines, common outputs such as activity minutes, EE, or recovery scores may represent different constructs across devices [11]. This raises questions of decision validity, creating a risk that researchers, clinicians, and users will synonymize proprietary estimates with direct physiological measurements or overinterpret small changes that may fall within expected measurement error. Future studies should therefore prioritize clear reporting of device models, firmware/software versions, data sources, wear-time criteria, missing-data handling, and analytic decisions.

Future progress will require stronger validation frameworks, greater transparency, and improved interoperability. Standardized definitions for commonly reported metrics, clearer documentation of algorithm changes, and reproducible data pipelines would improve cross-study comparison and evidence synthesis. Open or semi-transparent algorithms, when feasible, could help researchers better evaluate how sensor signals are transformed into physiological or behavioral estimates. For manufacturers, these efforts could increase user and researcher trust, support regulatory and clinical adoption, reduce ambiguity in external validation studies, and strengthen the long-term value of their platforms in health, research, and performance markets. In parallel, multi-device integration platforms and harmonized data infrastructures will be important for combining smartwatch data across brands, populations, and research settings.

AI-driven modeling and multimodal data fusion may further expand the utility of smartwatch data, particularly when combined with clinical, laboratory, environmental, and patient-reported information. However, these approaches must be developed carefully to avoid amplifying bias or errors, reducing interpretability, or generating decision-support tools that exceed the validity of the underlying data. Multimodal AI models may very well improve predictions, but they do not eliminate the need to establish whether the wearable inputs represent the true physiological construct. The future of smartwatch technology will depend not only on better sensors, but also on transparent algorithms, rigorous validation, equitable testing across diverse populations, and decision-support frameworks that align wearable outputs with meaningful health, rehabilitation, and performance outcomes.

## 8. Practical Recommendations for Using Smartwatch Data

The appropriate use of smartwatch-derived data begins with recognizing that consumer wearable outputs are not synonymous with gold-standard physiological measurements. Given the volume of data available from smartwatches, researchers, practitioners, and users should avoid allowing data availability alone to drive their questions and should consider what metrics are needed to investigate a specific question and limit analyses to parameters that are directly relevant to those questions.

In general, smartwatch data may be most useful for monitoring repeated measures and within-person trends over time. Changes in resting and exercise heart rate, daily physical activity habits, sleep duration and quality, or training loads may provide meaningful insight into behavior patterns and adaptation opportunities, even when absolute values contain some degree of measurement error. Greater caution is warranted when using smartwatch-derived metrics for cross-sectional comparisons between individuals, diagnostic classification, or precise quantification of complex physiological constructs such as EE, aerobic capacity, sleep, hydration status, or recovery readiness.

Selection and interpretation of smartwatch-derived outcomes should be guided by the intended population, activity type, and environment of use. Researchers, clinicians, and users should consider whether the device and outcome were evaluated under conditions similar to their intended application, such as steady-state walking, outdoor running, intermittent sport, rehabilitation, chronic disease management, or monitoring of older adults. When possible, devices should be selected based on evidence supporting the specific metric of interest in the relevant population and setting. When such evidence is unavailable, outputs should be interpreted cautiously and used primarily for exploratory or within-person trend monitoring rather than precise quantification or clinical decision-making.

Finally, smartwatch data should be integrated with, rather than substituted for, laboratory, clinical, or self-reported assessments. Wearables can provide ecological and longitudinal context that traditional assessments cannot capture, while laboratory and clinical measures remain essential for criterion-level assessment. The strongest applications of smartwatch technology are likely those in which wearable data complement established measures, helping researchers and practitioners understand how physiology, behavior, and performance unfold in daily life.

### Limitations

Several limitations of this review should be considered. First, as a narrative review, the literature identification and selection process was not exhaustive, and no formal systematic search, study-quality appraisal, or quantitative synthesis was performed. Accordingly, the included evidence may be influenced by publication availability, and the review should not be interpreted as providing pooled estimates of device validity. Second, the wearable technology literature is highly heterogeneous with respect to device brand and generation, firmware and algorithm version, reference standard, population, activity type, wear location, and environmental conditions, limiting direct comparison across studies. Third, proprietary algorithms and incomplete reporting often prevent full evaluation of how raw signals are transformed into user-facing outputs. Finally, smartwatch technology evolves rapidly, such that validation findings, regulatory status, platform access, and manufacturer documentation may change following publication. The conclusions should therefore be interpreted as a framework for critical evaluation rather than as a definitive ranking of devices or metrics.

## 9. Conclusions

Smartwatches are powerful tools for monitoring human health, physical activity, rehabilitation, and performance in real-world environments. Their broad accessibility, multifunctional design, widespread adoption, and capacity to collect continuous, longitudinal data distinguish smartwatches from many traditional monitoring tools and create opportunities to study health and performance in settings where individuals already engage with the technology. However, the value of smartwatch-derived data depends on understanding that many outputs are indirect estimates shaped by sensor quality, proprietary algorithms, user characteristics, device placement, and environmental context.

The central question is not simply whether a smartwatch can provide a given metric, but whether that metric is valid, interpretable, and appropriate for the intended purpose, population, and setting. Smartwatch-derived data may be especially useful for tracking within-person trends, characterizing habitual behavior, and complementing laboratory or clinical measures. However, caution is warranted when using these outputs for precise physiological quantification, diagnostic classification, or cross-device and cross-population comparisons.

As smartwatch technology continues to evolve, its impact in health and performance research will depend on addressing several core technical limitations, including motion-sensitive signal acquisition, constrained battery life and sampling frequency, limited access to raw data, proprietary and changing algorithms, inconsistent cross-device definitions, and reduced performance in underrepresented populations and uncontrolled environments. Advances in adaptive multisensor fusion, higher-precision positioning, transparent algorithms, and standardized data infrastructure may improve measurement continuity and ecological validity. These developments may also support under-explored applications such as passive monitoring of rehabilitation adherence, detection of meaningful deviations from individual baselines, integration of health and location data during emergency events, and context-aware monitoring across transitions between home, clinical, occupational, and outdoor environments. When used intentionally, smartwatches can move beyond passive monitoring to provide meaningful insight into how individuals behave, recover, and adapt in daily life. Their greatest promise lies not in measuring more, but in measuring what matters and interpreting those measurements responsibly.

## Figures and Tables

**Table 1:** Summary of smartwatch-derived metrics discussed in Section 4, including the underlying sensor or signal, criterion reference, overall validity, dominant confounding factors, and appropriate interpretive use.

Metric	Underlying Sensor/Signal	Criterion Standard	Validity Summary	Dominant Sources of Error	Appropriate Interpretation
Cardiovascular metrics: HR & SpO_2_	Optical PPG. HR is primarily estimated from reflected green-light PPG. SpO_2_ uses red and infrared wavelengths and ratio-of-ratios processing.	Multi-lead ECG for HR. Arterial blood gas or clinical co-oximetry for SpO_2_.	Across contemporary devices, resting and steady-state heart rate estimates commonly show MAE of ~2 bpm and MAPE below 10%; error generally increases during higher-motion conditions and rapid changes in exercise intensity. SpO_2_ estimates typically show RMSE of approximately 2–3% in normoxic ranges, with reduced reliability at lower oxygen saturations. Newer or higher-tier devices may reduce average error, but device tier alone does not ensure superior validity.	Motion artifact, poor device fit, sensor-skin contact, ambient light, tattoos or hair, exercise intensity, and individual differences in skin tone and tissue characteristics.	Useful for resting/steady-state trend monitoring and wellness-level screening cues. Not a substitute for clinical ECG or medical-grade oximetry.
Cardiovascular metrics: HRV	PPG derived pulse rate variability (PRV) from inter-pulse intervals; requires about 100–200 Hz sampling.	ECG-derived HRV (R–R intervals).	During resting or motionless recordings, PPG-derived PRV shows high agreement with ECG-derived HRV (ICC: 0.96–0.98). Sampling frequency affects error: with 100–200 Hz limiting PRV-index bias to below 2%, and sampling at 40–50 Hz increases bias beyond 20%. Agreement declines substantially during movement.	Motion artifact, peak-detection error, sensor sampling rate, and PPG-related effects of skin tone, tissue characteristics, age, sex, and fitness level.	Best interpreted as a within-person trend marker of autonomic balance during rest or sleep. Not appropriate as an ECG-HRV substitute for acute clinical interpretation.
Cardiovascular metrics: Single-Lead ECG	Electrical single-lead ECG using electrodes on the watch caseback and crown/side button.	Physician-interpreted 12-lead ECG.	Smartwatch single-lead ECG reports sensitivity and specificity above 90–95% for atrial-fibrillation discrimination under controlled conditions. One validation study reported 96% sensitivity and 100% specificity. Real-world performance is reduced by inconclusive tracings, motion, poor contact, and complex rhythms.	Motion during the recording, poor electrode contact, conduction abnormalities, paced rhythms, and age-related rhythm complexity	Useful for opportunistic atrial fibrillation screening and prompting clinical follow-up. Positive, negative, or inconclusive findings should be confirmed clinically when relevant.
EE & VO_2_max	Modeled estimates. EE (commonly from PPG-derived HR, accelerometer/gyroscope data, GPS, and anthropometric inputs) VO_2_max: (commonly from submaximal HR, GPS-derived pace, and anthropometric inputs).	Doubly labeled water or room calorimetry (EE)/ Graded exercise test with gas analysis (VO_2_max).	EE error frequently exceeds ±10–20%, and validation studies have not consistently demonstrated equivalence within ±10% across activities or devices. VO_2_max estimates generally show MAPE of approximately 5–13%, with better performance in untrained to moderately trained adults and larger errors among highly trained individuals.	Upstream sensor error (PPG, GPS, etc.), user-entered anthropometries, algorithmic unknowns, sex/age as equation terms, and fitness	Best used as a directional indicator of relative effort, not accurate calorie counts or absolute aerobic capacity. Reasonable for rough aerobic-fitness estimation and within-person change tracking.
Sleep & Readiness Scores	Sleep is inferred from actigraphy plus PPG-derived cardiac dynamics, sometimes supplemented by temperature or respiratory features. Readiness/recovery scores combine multiple inputs into proprietary composite scores.	Polysomnography (PSG) with EEG, EOG, and EMG for sleep staging. No established criterion standard exists for proprietary readiness scores.	Sleep-versus-wake detection generally shows sensitivity of at least 95% and overall agreement above 90% compared with PSG. Sleep-stage sensitivity is lower and varies by stage and device (50–86%), with a recurrent tendency to classify quiet wakefulness as light sleep. Proprietary readiness and recovery scores lack an established criterion standard and remain largely unvalidated as standalone constructs.	Insomnia, motionless awake periods, and PPG-related errors.	Useful for longitudinal sleep-duration and recovery-pattern monitoring. Not a substitute for PSG-based clinical sleep staging or validated clinical recovery assessment.
Body composition metrics	Small alternating current passed through the surface electrodes on the watch. Impedance is used to estimate total body water, fat-free mass, fat mass, and related outputs through regression models.	4-compartment model (most rigorous); DXA, MRI.	Smartwatch BIA demonstrates variable agreement with criterion body-composition methods, with reported MAPE of approximately 9–19%. Error is often proportional, increasing at higher body-fat percentages, and sex-specific differences in agreement have been reported. Performance is highly dependent on standardized hydration and measurement conditions.	Hydration level, electrode contact, body fat percentage, and sex/age as equation terms.	Most appropriate for standardized within-person trends. Not a precise single-measure replacement for criterion body fat or skeletal muscle percentage.
Mechanics: Spatiotemporal, Distance, Velocity, and Movement Metrics	IMU signals from accelerometers, gyroscopes, and magnetometers combined with GPS when available.	Surveyed distance, timing gates, radar, GPS reference systems, or 3D motion capture with force plates.	Step count and average cadence generally show the strongest agreement, with step-count error often below 10% during normal-speed outdoor walking. More complex wrist-derived running metrics show substantially poorer agreement; reported MAPE values for stride length and ground-contact time are approximately 15–19%, with concordance coefficients near 0.30–0.39. Performance varies by model, wear location, activity, and GPS environment.	Wear location, wrist-arm motion coupling, gait speed, age- or pathology-related gait changes, running mechanics, GPS obstruction, and indoor/indoor-adjacent environments.	Appropriate for steps, cadence, and outdoor distance/pace under favorable conditions. Complex gait mechanics should be interpreted cautiously, especially from wrist-only devices.
Emerging metrics: Cuffless BP	Algorithmic estimate from PPG, PAT and individual cuff calibration.	Validated cuff-based sphygmomanometry or invasive intra-arterial pressure in controlled studies.	Some studies report MAE near the commonly cited 5-mmHg threshold, but agreement is highly variable. In one intensive-care validation, approximately 50–85% of estimates fell within 5 mmHg of the reference value, depending on the pressure outcome and condition. Accuracy is greatest soon after calibration and may deteriorate with physiologic change. Expert guidance continues to recommend caution for clinical use.	Calibration interval and quality, PPG-related errors, and posture, exercise, sleep, medication effects.	Early/pre-clinical. Should not be used for hypertension diagnosis or management unless supported by appropriate regulatory clearance and clinical validation for that use.
Emerging metrics: Sweat loss/hydration	Modeled estimates from HR, skin temperature, exercise intensity, accelerometry, ambient temperature, and humidity.	Whole-body mass change for sweat loss, laboratory electrolyte analysis for hydration/sweat composition.	Available smartwatch validation studies report large errors, with MAPE of approximately 25–33% relative to whole-body sweat-loss criteria. Agreement varies by activity, environment, fitness level, and sex and differs between devices. Current smartwatch outputs remain modeled estimates rather than direct sweat or fluid-balance measurements.	Exercise mode and intensity, environment, fitness level, sex, and absence of direct sweat sampling in current smartwatch platforms.	Best treated as approximate field estimates or prompts for hydration planning. Not a direct measurement of sweat chemistry, dehydration, or whole-body fluid balance.
Emerging metrics: Non-invasive glucose monitoring	Developmental approaches include optical PPG or spectroscopy-based signals.	Laboratory plasma glucose, minimally invasive CGM serves as the current practical clinical benchmark.	No smartwatch has been authorized for direct non-invasive glucose measurement, and available optical or spectroscopic approaches remain developmental. Accuracy has not consistently approached that of minimally invasive continuous glucose monitors, which commonly achieve mean absolute relative differences of approximately 8–10%.	Weak glucose signal relative to water, hemoglobin, protein, and fat; skin thickness, hydration, temperature, perfusion, pigmentation, calibration drift, and limited generalizability across individuals.	Not appropriate for clinical glucose monitoring. Watch displays may be useful only when relaying data from an authorized CGM system.

Abbreviations: BIA, bioelectrical impedance analysis; BP, blood pressure; CGM, continuous glucose monitor; DXA, dual-energy X-ray absorptiometry; ECG, electrocardiography; EE, energy expenditure; EEG, electroencephalography; EMG, electromyography; EOG, electrooculography; GPS, global positioning system; HR, heart rate; HRV, heart rate variability; ICC, intraclass correlation coefficient; IMU, inertial measurement unit; MAE, mean absolute error; MAPE, mean absolute percentage error; MRI, magnetic resonance imaging; PAT, pulse arrival time; PPG, photoplethysmography; PRV, pulse rate variability; PSG, polysomnography; RMSE, root mean square error; SpO_2_, peripheral oxygen saturation; VO_2_max, maximal oxygen uptake.

## Data Availability

No new data were created or analyzed in this study. Data sharing is not applicable to this article.

## Abbreviations

The following abbreviations are used in this manuscript:

GPS	Global Positioning System
PPG	Photoplethysmography
IMU	Inertial measurement unit
LED	Light-emitting diode
HRV	Heart rate variability
SpO_2_	Peripheral oxygen saturation
ECG	Electrocardiography/electrocardiogram
MAPE	Mean absolute percentage error
FDA	U.S. Food and Drug Administration
PRV	Pulse rate variability
VO_2_max	Maximal oxygen uptake
EE	Energy expenditure
MET	Metabolic equivalent of task
BMR	Basal metabolic rate
PSG	Polysomnography
REM	Rapid eye movement
EEG	Electroencephalography
EOG	Electrooculography
EMG	Electromyography
BIA	Bioelectrical impedance analysis
BP	Blood pressure
PTT	Pulse transit time
PAT	Pulse arrival time
SDK	Software development kit
API	Application programming interface
